# Hematopoietic chimerism after allogeneic stem cell transplantation: a comparison of quantitative analysis by automated DNA sizing and fluorescent *in situ *hybridization

**DOI:** 10.1186/1471-2326-5-1

**Published:** 2005-01-10

**Authors:** Justyna Jółkowska, Anna Pieczonka, Tomasz Strabel, Dariusz Boruczkowski, Jacek Wachowiak, Peter Bader, Michał Witt

**Affiliations:** 1 Division of Molecular and Clinical Genetics, Institute of Human Genetics, Strzeszyñska 32, 60-479 Poznañ, Poland; 2 Department of Pediatric Oncology, Hematology and Transplantology, University of Medical Sciences, Szpitalna 27/33, 60-572 Poznañ, Poland; 3 University of Agriculture, Department of Genetics and Animal Breeding, Wołyñska 33, 60-627 Poznañ, Poland; 4 University Children Hospital III MRD-/ Chimerism Laboratory, Frankfurt/Main, Germany (University Children Hospital Tuebingen previously); 5 International Institute of Molecular and Cell Biology, Trojdena 4, Warszawa, Poland

## Abstract

**Background:**

Allogeneic hematopoietic stem cell transplantation (allo-HSCT) is performed mainly in patients with high-risk or advanced hematologic malignancies and congenital or acquired aplastic anemias. In the context of the significant risk of graft failure after allo-HSCT from alternative donors and the risk of relapse in recipients transplanted for malignancy, the precise monitoring of posttransplant hematopoietic chimerism is of utmost interest. Useful molecular methods for chimerism quantification after allogeneic transplantation, aimed at distinguishing precisely between donor's and recipient's cells, are PCR-based analyses of polymorphic DNA markers. Such analyses can be performed regardless of donor's and recipient's sex. Additionally, in patients after sex-mismatched allo-HSCT, fluorescent *in situ *hybridization (FISH) can be applied.

**Methods:**

We compared different techniques for analysis of posttransplant chimerism, namely FISH and PCR-based molecular methods with automated detection of fluorescent products in an ALFExpress DNA Sequencer (Pharmacia) or ABI 310 Genetic Analyzer (PE). We used Spearman correlation test.

**Results:**

We have found high correlation between results obtained from the PCR/ALF Express and PCR/ABI 310 Genetic Analyzer. Lower, but still positive correlations were found between results of FISH technique and results obtained using automated DNA sizing technology.

**Conclusions:**

All the methods applied enable a rapid and accurate detection of post-HSCT chimerism.

## Background

Allogeneic hematopoietic stem cell transplantation (allo-HSCT) is performed mainly in patients with high-risk or advanced hematologic malignancies and aplastic anemias, and for some of them it is the only curative treatment. After allo-HSCT, incomplete engraftment and appearance of recipient's hematopoietic cells can lead to a coexistence of donor and host hematopoiesis – a situation known as mixed chimerism. Complete recovery of hematopoiesis of the donor origin is referred to as complete chimerism.

Widely accepted molecular methods for analysis of chimerism after allo-HSCT, aimed at distinguishing precisely between donor's and recipient's cells, are PCR-based analyses of polymorphic DNA markers, such as variable number of tandem repeats (VNTR) or short tandem repeats (STR) [1]. Such analyses can be performed regardless of donor's and recipient's sex [2]. Additionally, in patients after sex-mismatched allo-HSCT, fluorescent *in situ *hybridization (FISH) can be applied. This technique is based on identification of Y-chromosome-specific sequences in the posttransplant sample examined. Variants of these techniques can be used for precise, quantitative assessment of the amount of donor's cells in recipient's peripheral blood and/or bone marrow after transplantation, in the long run giving a picture of the dynamics of changes in chimeric status within a hematopoietic compartment. Chimerism also reflects response to treatment [3], since it correlates with the risk of malignancy relapse. Relapse is the most frequent cause of treatment failure in recipients transplanted for hematologic malignancies, but it is still controversial if patients with mixed chimerism have an increased risk of developing relapse or graft failure. Most probably only a progressive mixed chimerism (a dynamic rise in the number of recipient's cells over time) seems to reflect a relapse or rejection. Successful outcome has been associated with a state of stable complete chimerism [4].

Here we report results of a comparison of different techniques for analysis of posttransplant chimerism: fluorescent *in situ *hybridization (FISH) and PCR-based molecular methods with fluorescent products detected in an ALF Express DNA Sequencer (Pharmacia) or ABI 310 Genetic Analyzer (PE).

## Methods

### Patients

Investigation of hematopoietic chimerism was performed in ten children (eight girls, two boys) aged 6–16 years. They were diagnosed with acute myelogenous leukemia (n = 4), acute lymphoblastic leukemia (n = 2), chronic myelogenous leukemia (n = 1), myelodysplastic syndrome (n = 1), or Fanconi Anemia (n = 2). All recipients received hematopoietic stem cells from HLA-matched sibling donors, and sex-mismatch between donor and recipient was present in all cases. The material (peripheral blood) for hematopoietic chimerism quantification was collected in different periods after transplantation. In all 10 children reconstitution of hematopoiesis was observed. Out of 8 children transplanted for hematologic malignancies, 4 are well and alive, in complete continuous remission (CCR), while in the other 4, leukemia relapse occurred 4–23 months after transplantation.

### DNA isolation

High-molecular-weight DNA was extracted from frozen whole blood (approximately 5 ml) or bone marrow (approximately 3–5 ml) by the standard treatment with sodium dodecyl sulfate (SDS) and proteinase K, and the salting-out method. DNA was isolated from the donors' and patients' blood samples collected before and after transplantation at various intervals in order to determine the chimeric status.

### Analysis of PCR products by an ABI 310 Genetic Analyzer

The PCR protocol optimized for the Qiagen polymerase and the PE 9700 thermocycler was performed as described previously [5]. For fragment analysis (after capillary electrophoresis), an ABI 310 Genetic Analyzer (PE) was used [5]. All analyses were performed in the University Children's Hospital, Tuebingen.

### Analysis of PCR products by an ALF Express DNA Sequencer

The PCR protocol optimized for the Thermal Controller MJ Research (Watertown, MA) model PTC-100™ was applied. PCR was performed in a volume of 10 μl; the PCR reaction mixture contained: 2.5 pM of each forward and reverse primer, 200 μl of each dNTP, 0.4 U Taq polymerase (Qiagen, Chatsworth, CA), 1x PCR buffer (Qiagen, Chatsworth, CA), and 40 ng of genomic DNA. Conditions for PCR were as follows: 5 min at 94°C for the first denaturation; 26 cycles of amplification with a temperature profile of 45 sec at 94°C, 1 min at 55°C, 1 min at 72°C; with additional 5 min at 72°C in the last cycle. STR loci were amplified with fluorescent PCR primers described previously [6]. Primers for microsatellite markers were labeled with Cy5 dye (TIB MOLBIOL). A 1.5-μl aliquot of PCR reaction was resuspended in 7 μl of loading solution (formamide, bromophenol blue) containing 100 bp and 300 bp internal markers. All samples, after denaturation at 95°C for 5 min, were analyzed on 6% denaturing polyacrylamide gel with 7 M urea in the sequencer. A 50–500 sizer labeled with Cy5 dye was used as an external marker (for calculation of allele sizes). Electrophoresis was carried out in 0.6xTBE buffer at 1500 V/min. The helium-neon laser was operated at a wavelength of approximately 700 nm and laser power value of 2.5 mW. Allele sizes and peak areas of fluorescent products were analyzed and calculated with the use of Fragment Manager software (Pharmacia). PCR and analysis of PCR products by the ALF Express DNA Sequencer was performed at the Institute of Human Genetics, Poznan.

### FISH

The experiments were performed on interphase nuclei obtained by standard short culturing of fresh whole blood samples, with probes specific for chromosomes X (locus *DXZ1*) and Y (locus *DYZ1*). The FISH procedure according to Cytocell Ltd. was used [7]. The number of scored nuclei was 250 to 550, with a median of 300. FISH experiments were performed at the Institute of Human Genetics, Poznan. Since the material for FISH analysis was not collected in all designated periods after transplantation in some patients, FISH experiments were not performed then.

### Quantification of chimerism

After electrophoresis in the ABI 310 Genetic Analyzer (PE), all obtained data were analyzed by GeneScan 3.1 software and then transferred to Genotyper 2.5 software [5]. All data obtained after electrophoresis of fluorescent products in the ALFExpress DNA Sequencer were transferred to Fragment Manager™ software (Pharmacia). For both, calculation of the amount of recipient's DNA was performed using the formula:

% of recipient's DNA = (R1 + R2)/(D1+ D2 + R1 + R2) × 100,

where: R1, R2 = peak areas of recipient's alleles; and D1, D2 = peak areas of donor's alleles.

Only informative markers were used for the analysis. If donor and recipient were heterozygous but shared one allele, only the area of the non-shared alleles was considered for the analysis [8].

To make sure that quantification is accurate, we performed serial dilution experiments, where standardized mixed chimeric samples were created by mixing donor's and pretransplant recipient's DNA in a range between 0 and 100 percent. The sensitivity strongly depends on the size of alleles, the detection level was around 3–5% of patient cells.

The results of chimerism detection by different methods were compared by the Spearman correlation test.

## Results

The results of chimerism quantification with the use of an ALF Express DNA Sequencer, ABI 310 Genetic Analyzer, and FISH are compared in Table 1. In three patients (no. 5, 6, 7) only donor's cells were detected in all post-HSCT samples and in one patient (no. 1) only recipient's cells were present in all samples examined. These results were confirmed with the use of all three methods. Three other patients (no. 2, 3, 4) exhibited mixed posttransplant chimerism according to PCR and/or FISH. In the last three patients (no.8, 9, 10), complete chimerism was detected by PCR and automated DNA sizing, but in some of their samples, low numbers of recipients' cells were detected by FISH.

**Table 1:** Comparison of results using different DNA sizing technologies and FISH

**No.**	**UPN**	**Sex F/M**	**Diagnosis**	**Material examined**	**HSCT date**	**Days after HSCT**	PCR/ALF Express*	PCR/ ABI 310 Genetic Analyser*	FISH with probes specific to X, Y chro-mosomes*
1.	67	F	AML	PB	04.09.1998	542	100%	100%	100%
						556	100%	100%	100%
						574	100%	100%	100%
						590	100%	100%	100%
						639	100%	100%	100%
						675	100%	100%	100%
						697	100%	100%	100%
						721	100%	100%	100%
						750	100%	100%	100%
2.	102	M	AML	PB	12.10.2000	14	84%	86%	89%
						19	49%	47%	51%
						28	40%	40%	35%
						35	47%	42%	12%
						57	46%	35%	6%
3.	106	M	FA	PB	21.12.2000	7	100%	100%	92%
						48	26%	27%	32%
						91	13%	14%	9%
4.	87	F	FA	PB	27.12.1999	21	10%	28%	ND
						38	93%	94%	ND
						285	15%	18%	ND
						313	17%	19%	ND
						357	0%	0%	ND
5.	45	F	CML	PB	15.11.1996	953	0%	0%	ND
						1030	0%	0%	ND
						1216	0%	0%	ND
						1284	0%	0%	0%
						1374	0%	0%	0%
						1459	0%	0%	0%
						1492	0%	0%	ND
6.	93	F	AML	PB	20.04.2000	28	0%	0%	0%
						158	0%	0%	0%
						277	0%	0%	0%
7.	109	F	MDS	PB	26.01.2001	14	0%	0%	0%
						21	0%	0%	ND
						34	0%	0%	ND
						42	0%	0%	ND
8.	108	F	ALL	PB	19.01.2001	17	0%	0%	ND
						21	0%	0%	ND
						26	0%	0%	2%
						39	0%	0%	ND
						52	0%	0%	ND
						60	0%	0%	ND
9.	88	F	AML	PB	21.01.2000	29	0%	0%	ND
						87	0%	0%	0%
						178	0%	0%	ND
						267	0%	0%	ND
						365	0%	0%	2%
10.	95	F	ALL	PB	16.06.2000	27	0%	0%	ND
						83	0%	0%	ND
						200	0%	0%	ND
						218	0%	0%	1%

ALL = acute lymphoblastic leukemia; AML = acute myelogenous leukemia; CML = chronic myelogenous leukemia; FA = Fanconi Anemia; FISH = fluorescent *in situ *hybridization; HSCT = hematopoietic stem cell transplantation; MDS = myelodysplastic syndrome; ND = no data; PB = peripheral blood; UPN = unique patient's number

* results expressed as % of recipient's cells

Coefficients of Spearman rank correlation between results of chimerism quantification by the three different methods are shown in Table 2. All coefficients were statistically significant (p < 0.001). The correlation between PCR/ALF Express and PCR/ABI 310 was stronger than between FISH and both PCR methods.

**Table 2:** Coefficients of Spearman rank correlation between results of chimerism quantification by different methods (all coefficients significant, P < 0.001).

	**PCR/ABI 310 Genetic Analyzer**	**FISH**
PCR/ALF Express	0.987	0.801
PCR/ABI 310 Genetic Analyzer		0.825

### Comparison of methods used

One of the advantages of application of automated DNA sizing techniques for detection of posttransplant chimerism is that the use of radioactivity is not necessary. It is arelatively simple and rapid method, consisting of two steps: polymerase chain reaction with fluorescent primers and automated detection of fluorescently labeled PCR products, separated by electrophoresis. The analysis of fluorescently labeled PCR products provides better accuracy and precision of measurement than traditional electrophoretic methods. The most time-consuming step, which might prolong the examination process, is the search for informative markers.

Analysis of the chimerism status by amplification of STR loci can be performed regardless of donor's and recipient's sex. The most advantageous is the high sensitivity of the detection system used, so that only small amounts of DNA are needed.

Fragment analysis in the ABI 310 Genetic Analyzer (PE) is performed after capillary electrophoresis. The examination of one sample requires about 6 hrs; 144 samples can be analyzed per day if three colors are simultaneously analyzed [5]. The advantage of this method is that thanks to the combination of three-color detection, multiple sets of samples or multiple loci for a single sample can be analyzed on one gel. It is possible to analyze more than one marker at a time after multiplex amplification of informative loci.

In the ALFExpress DNA Analyzer, electrophoresis is carried out in an off-vertical gel cassette specially designed for easy and safe gel casting. The number of samples to be loaded on a gel is limited to 40 per run. The ALFwin Fragment Analyzer is used afterwards for fragment analysis. It is provided with versatile application software for the control of DNA fragment separation runs and subsequent analysis of the data. Collected data are used to accurately size PCR product peaks on the basis of external and internal standards.

One analysis of 40 samples takes 7 hrs, including PCR, electrophoresis in the gel cassette, and paperwork. The appropriate assembling and cleaning of the gel cassette is critical and time consuming.

It is well known that FISH is a good quantitative method of fluorescent signal detection, but requires lots of technical experience and expertise. Fluorescent *in situ *hybridization for one patient sample lasts at least 5 hrs, including preparation of interphase nuclei, hybridization with specific probes (X, Y dual-color FISH), and analysis. The high cost of the procedure is definitely a disadvantage.

## Discussion

PCR-based techniques allow the relative proportions of recipient's and donor's cells in the post-HSCT period to be identified and quantified and is not only limited to sex-mismatched transplants. Although when using chimerism analysis one cannot assess whether or not the population of recipient's nucleated cells contains leukemic cells, samples taken at various intervals can show if the expansion rate of the particular population is consistent with hematologic and clinical symptoms of the disease. When it is not possible to find an informative marker for PCR amplification, only FISH analysis enables assessing the chimerism status. However, cytogenetic Y chromosome probing by FISH is limited exclusively to sex-mismatched transplantations. Results obtained with the use of ALFExpress DNA Sequencer and ABI 310 Genetic Analyzer are identical or very similar. We showed that appropriate quantitative assessment of chimerism after HSCT by using microsatellite genotyping and automated DNA sizing does not depend on the sequencer model used. The high correlation between results from the PCR/ALFExpress and PCR/ABI 310 Genetic Analyzer indicate that these two methods can be used interchangeably. The superiority of the ABI 310 Genetic Analyzer is limited to the possibility of analysis of three samples at the same time in one reaction tube and the technical ease of capillary electrophoresis with no need for the time-consuming and cumbersome use of glass plates. Lower, but still positive correlations were found between results of FISH analysis and these two methodological variants of PCR. However, in some samples analyzed with PCR, no recipient's signals were found, attesting to full donor chimerism, while at the same time residual host cells turned out to be detectable by FISH. We suggest that these results are within the range of error of the method applied.

## Conclusions

Finally, we conclude that all the methods applied enable a rapid and accurate detection of post-HSCT chimerism and with due caution can be used interchangeably.

## Competing interests

The author(s) declare that they have no competing interests.

## Authors' contributions

J.J. carried out the molecular chimerism studies and drafted the manuscript.

T.S. performed the statistical analysis.

A.P and D.B. supplied clinical data.

P.B. initiated quantitative analysis.

J.W. supervised clinical part and final writing

M.W. supervised laboratory part and final writing

## Pre-publication history

The pre-publication history for this paper can be accessed here:


